# Early Mobilization Compliance as a Quality Indicator After Hip Fracture Surgery: An Observational Study

**DOI:** 10.3390/jcm15093377

**Published:** 2026-04-28

**Authors:** Nelly Esperanza Endara-Tello, Miriam Batalla-Pascua, Silvia Córdoba-Ortega, Miriam Álvarez-Villarreal, Francisco Javier García-Sánchez

**Affiliations:** 1 Surgical Hospitalization Unit, Hospital Universitario Infanta Cristina, Instituto de Investigación Sanitaria Hospital Puerta de Hierro Segovia Arana (IDIPHISA), 28981 Madrid, Spain; nellyesperanza.endara@salud.madrid.org (N.E.E.-T.); miriam.batalla@salud.madrid.org (M.B.-P.); silvia.cordobao@salud.madrid.org (S.C.-O.); 2 Research Nursing Unit, Hospital Universitario Infanta Cristina, Instituto de Investigación Sanitaria Hospital Puerta de Hierro Segovia Arana (IDIPHISA), 28981 Madrid, Spain; 3 Emergency Room Service, Surgical Prehabilitation Unit, Hospital Universitario Infanta Cristina, Instituto de Investigación Sanitaria Hospital Puerta de Hierro Segovia Arana (IDIPHISA), 28981 Madrid, Spain; franci11@ucm.es; 4 Medical Department, Faculty of Medicine, University Complutense of Madrid, 28040 Madrid, Spain

**Keywords:** hip fracture, hospitalization, early mobilization, quality indicator

## Abstract

**Background**: Hip fracture represents a major public health challenge in aging populations and is associated with high morbidity, functional decline, and increased healthcare utilization. Early mobilization within the first 24 h after surgery is considered a key quality indicator in hip fracture care and has been associated with improved functional recovery and reduced postoperative complications. The aim of this study was to evaluate compliance with early mobilization and identify clinical and organizational factors associated with its implementation. **Methods**: A retrospective observational study was conducted in the surgical hospitalization unit of a tertiary hospital in Madrid, Spain. All adult patients who underwent hip fracture surgery between January and December 2023 were included. Sociodemographic, clinical, and functional variables were collected from electronic medical records. Early mobilization within the first 24 h after surgery was defined as the primary outcome and analyzed in relation to clinical and organizational variables. **Results**: A total of 139 patients were included, with a mean age of 82.4 ± 11.3 years, and 79.1% were women. Early mobilization was achieved in 66.2% of patients. Significant associations were observed between early mobilization and blood transfusion requirements (*p* = 0.002), postoperative radiography performed within the first 24 h (*p* = 0.004), and the presence of an explicit medical order for mobilization (*p* < 0.001). No significant associations were identified with age, sex, functional dependency, cognitive impairment, baseline mobility, or ASA classification. **Conclusions**: Early mobilization after hip fracture surgery was associated with several modifiable clinical and organizational factors in this cohort. However, these findings should be interpreted with caution because fracture-specific and surgery-specific variables were not available. Implementing standardized protocols, automatic mobilization orders, and optimized perioperative management strategies may improve compliance with this key quality indicator and enhance postoperative recovery.

## 1. Introduction

Hip fractures represent a major epidemiological problem, strongly associated with population aging [1]. In Spain, according to the latest available National Hip Fracture Registry (Registro Nacional de Fracturas de Cadera, RNFC) report published in 2025, which includes data from patients ≥74 years treated in collaborating Spanish hospitals during 2023, a total of 9906 cases were recorded. The Community of Madrid ranks among the regions contributing the highest volume of data, accounting for approximately 30% of national cases in the analyzed years. The average hospital stay is slightly above the national mean, whereas surgical timing, in-hospital mortality, and 30-day mortality remain comparable [2].

Since its establishment in 2017, the RNFC has defined eight healthcare quality indicators aimed at monitoring hospital performance and benchmarking best practices based on top-performing centers. Among these indicators, early sitting or mobilization within the first 24 h after surgery is considered a key parameter for early functional recovery [3]. In addition, international clinical guidelines and care pathways for hip fracture management recommend early mobilization as soon as clinically feasible, ideally on the day of surgery or within the first 24 h. This recommendation is based on evidence showing reductions in postoperative complications, improved functional recovery, and shorter hospital length of stay.

The evolution of the early mobilization indicator (less than 24 h) in the Community of Madrid is available from 2017 to 2020, with reported rates of 59.2%, 54.8%, 40.3%, and 12.4%, respectively. These figures remained similar to or higher than the national average (58.9%, 58.5%, 38%, and 11%), with a marked decline in 2020 associated with the impact of the COVID-19 pandemic. The most recent registry data (2023) show a favorable recovery trend in 2021, 2022, and 2023 (72.3%, 75%, and 75.8%), although still below the recommended standard of 86% [2].

Early mobilization after hip fracture surgery has been associated with improved short-term functional recovery, reduced postoperative pain, greater restoration of baseline functional capacity, shorter length of stay (LOS), improved quality of life, and higher survival rates [4]. This early mobilization requires coordinated collaboration among surgeons, therapists, and nursing staff to facilitate weight-bearing mobilization on the day of surgery or the first postoperative day, aiming to prevent complications and accelerate rehabilitation [5].

The coordinated work of multidisciplinary healthcare professionals is, therefore, essential and requires a comprehensive approach that includes surgical planning and the adaptation of health and social care resources [6]. Training, role definition, and competency development are key factors in achieving a truly multidisciplinary model that enables patients to get out of bed on the first postoperative day [7].

Barriers to mobilization include non-modifiable patient-related factors such as age, pre-fracture mobility, and cognitive impairment [8], as well as system-related factors including staffing availability, resource constraints, healthcare professionals’ attitudes toward early mobilization, and role delineation in mobilization practices [9,10].

While surgical factors such as fracture type, fixation stability, and weight-bearing protocols may influence mobilization, less is known about the impact of modifiable organizational and clinical processes in routine care.

In this context, our hospital, as a collaborating center of the RNFC, annually evaluates several quality indicators, including the proportion of patients mobilized on the first postoperative day. In 2023, compliance with this indicator decreased compared to the previous year, prompting the need to describe and analyze the causes of non-compliance.

Therefore, the aim of this study was to identify the determinants of non-compliance and to analyze the associated clinical and/or organizational factors, with the purpose of proposing and implementing mechanisms to improve this quality indicator.

## 2. Materials and Methods

### 2.1. Study Design and Setting

This observational, analytical, and descriptive retrospective study was conducted in the surgical hospitalization unit of Hospital Universitario Infanta Cristina, Madrid (Spain), between 1 January 2023, and 31 December 2023.

The study population included all adult patients who underwent surgery for hip fractures. For the purpose of this study, hip fracture was defined as a proximal femoral fracture requiring surgical treatment and admission to the surgical hospitalization unit. Due to the retrospective design and limitations in the structured availability of surgical data, fracture subtype (e.g., femoral neck versus trochanteric), surgical technique (internal fixation versus arthroplasty), implant selection, surgical approach, and postoperative weight-bearing protocols were not consistently available and were therefore not included in the present analysis. Patients who died within the first 24 h after surgery were excluded.

### 2.2. Sample Size

The sample size was estimated for the primary outcome—compliance with early mobilization within the first 24 h after hip fracture surgery—using the standard formula for estimating a single proportion with a specified absolute precision: $n=Z_{1-\alpha /2}^{2}\;p\left(1-p\right)/d^{2}$. An expected compliance proportion of p=0.75 was assumed, based on the national compliance rate reported by the Spanish National Hip Fracture Registry (RNFC) for 2021–2022, which was the most recent benchmark available at the time the study was designed [2].

With a two-sided 95% confidence level ($Z_{1-\alpha /2}=1.96$) and an absolute precision of d=±6.8 percentage points, the minimum required sample size was 156 patients. This precision was considered clinically acceptable given the unicentric design and the expected annual surgical volume of the participating unit. The final analytical sample comprised 139 patients after the exclusion of records with incomplete data for the primary outcome or for key covariates of the multivariable model; this is acknowledged as a limitation and is addressed in the Discussion.

Data were retrospectively obtained through a review of electronic medical records (SELENE system). For each patient, an ad hoc data collection form was completed, and the information was subsequently entered into a database specifically created for the study.

Sociodemographic variables included age (<69 years, 70–84 years, and ≥85 years) and sex.

Additional clinical and functional data were collected, including comorbidities, admission dates, blood transfusion requirements, the performance of postoperative radiography within 24 h, and the existence of an explicit medical order for early mobilization.

To define preoperative functional autonomy, the Barthel Index was used to assess independence in activities of daily living (ADL), the Pfeiffer Scale to evaluate cognitive status, and the Functional Ambulation Classification (FAC) scale to assess baseline mobility.

Preoperative physical status and anesthetic risk were described using the American Society of Anesthesiologists (ASA) classification.

Finally, mobilization and/or sitting up in bed within the first 24 h after surgery was recorded.

### 2.3. Statistical Analysis

A multivariable binary logistic regression model was fitted to identify factors independently associated with early mobilization within the first 24 h after surgery. Candidate covariates were pre-specified on the basis of clinical and biological plausibility, drawing on previously reported determinants of early mobilization after hip fracture surgery [8,9,10], rather than on statistical significance in bivariate screening, in order to avoid data-driven variable selection in a sample of limited size. The following variables were included in the model: age (continuous, in years), ASA physical status classification (categorical, with ASA I as reference), baseline mobility status according to the adapted Functional Ambulation Classification (categorical, with “independent” as reference), and the presence of an explicit medical order for mobilization (dichotomous). All candidate covariates were entered simultaneously into the model using the enter (forced-entry) method; no stepwise, forward, or backward automated selection procedures were applied, in line with current recommendations that discourage automated selection algorithms in confirmatory clinical analyses with a limited number of events [11]. To minimize the risk of overfitting, the number of predictors was kept low, respecting the commonly cited rule of at least 10 events per variable relative to the less frequent outcome category (n = 47 non-mobilized patients). Results were reported as adjusted odds ratios (aOR) with 95% confidence intervals and corresponding *p*-values. All analyzes were performed using IBM SPSS Statistics^®^ version 25.

### 2.4. Ethical Aspects

The study was approved (PI 149/24) by the Research Ethics Committee of Hospital Universitario Puerta de Hierro Majadahonda. The study was conducted in accordance with the Declaration of Helsinki.

## 3. Results

### 3.1. Participant Demographic Data

During the study period, a total of 139 patients underwent hip fracture surgery. Of these, 79.1% (n = 110) were women and 20.9% (n = 29) were men. The mean age of the patients was 82.4 years (SD 11.3; range: 31–101 years). Although the study included patients across a wide age range, the majority corresponded to older adults with fragility fractures. A small proportion of younger patients were included, reflecting cases managed within the same clinical pathway. This heterogeneity should be considered when interpreting the results.

### 3.2. Functional and Mobility Levels

Functional and autonomy levels are shown in Table 1.

According to the Barthel Index, more than half of the participants were independent in activities of daily living (51.8%, 72/139).

Consistent with these findings, the Pfeiffer Scale showed that the same proportion of patients presented no signs of cognitive impairment (51.8%).

Regarding mobility, based on the Functional Ambulation Classification (FAC) scale—adapted into the categories: independent, walks with difficulty, walks with technical assistance, walks with major assistance or great difficulty, and bed–chair bound—approximately one-third of patients (33.1%, 46/139) required some type of assistive device, and more than half showed mobility limitations (55.4%, 77/139).

### 3.3. ASA Classification System

According to the American Society of Anesthesiologists (ASA) physical status classification, which stratifies morbidity and mortality risk in patients undergoing anesthesia and surgery, patients are categorized from ASA I to ASA V.

ASA I corresponds to patients fit for any standard anesthetic procedure, with minimal anesthetic risk and a low probability of anesthesia-related complications. Conversely, ASA V represents critically ill, moribund patients who are not expected to survive without surgery.

Most patients presented a highly compromised clinical profile characterized by a high burden of disease and considerable anesthetic risk.

A total of 65.9% of participants were classified as ASA III, corresponding to patients with one or more severe systemic diseases causing significant functional limitations.

This finding is consistent with the typical profile of pluripathological patients in medium- and high-complexity hospital settings, where comorbidities directly impact anesthetic risk and perioperative planning.

Additionally, 18.1% of patients were classified as ASA II, indicating mild or well-controlled systemic disease without relevant functional limitation.

Only 2.2% were categorized as ASA I.

Regarding more severe categories, 13.0% were classified as ASA IV, representing patients with severe systemic disease, disabling functional limitations, and life-threatening risks prior to surgery, requiring specialized perioperative management.

Finally, 0.7% were classified as ASA V.

ASA classification data were available for 138 of the 139 patients included in the study.

### 3.4. Early Mobilization

Early mobilization within the first 24 h after surgery was analyzed.

Results showed that 66.2% of patients (n = 92) were mobilized within the first 24 h, whereas 33.8% (n = 47) did not receive early mobilization.

This observed rate suggests moderate compliance with early mobilization recommendations while highlighting a substantial proportion of patients who were not mobilized early.

The determinants of early mobilization are presented in Table 2.

#### Reasons for Lack of Early Mobilization

When analyzing the reasons for non-mobilization recorded in nursing progress notes, among the 47 non-mobilized patients, no cause was documented in 29.7% of cases (n = 12).

For the remaining cases, reasons were grouped thematically due to variability in narrative documentation.

Five main categories were identified:

- Pending radiography/medical order or delayed prescription: 17.0% (n = 10)
- Decreased level of consciousness, somnolence, or hypoactive status: 14.9% (n = 7)
- Hypotension or blood transfusion requirements: 12.8% (n = 6)
- Agitation, hyperactive status, need for restraints, or medical indication for mobilization at 48 h: 10.7% (n = 5).
- Patient refusal or lack of collaboration: 4.2% (n = 2)

### 3.5. Bivariate Analysis

Bivariate analysis showed no statistically significant associations between mobilization within the first 24 h and the sociodemographic or clinical variables evaluated.

No significant differences were observed by sex (*p* = 0.093) or age (*p* = 0.139), although a trend toward lower mobilization rates was identified among older patients.

Similarly, language barriers did not reach statistical significance (*p* = 0.077); however, a clinically relevant difference was observed between patients with language barriers (20% mobilized) and those without (67.9%), suggesting a potential influence on patient participation in postoperative activities.

No significant associations were found with functional dependency (*p* = 0.620), cognitive impairment (*p* = 0.158), baseline mobility (*p* = 0.334), or ASA classification (*p* = 0.431).

### 3.6. Multivariable Analysis

A multivariable binary logistic regression model was fitted, with early mobilization within the first 24 h as the dependent variable, including age (continuous), ASA physical status classification, baseline mobility status (adapted FAC), and the presence of an explicit medical order for mobilization as independent variables, entered simultaneously using the forced-entry (enter) method. The model was estimated on the 139 patients included in the analytical sample (92 mobilized, 47 non-mobilized).

In the adjusted model, the presence of an explicit medical order for mobilization was the only variable independently and significantly associated with early mobilization, with an adjusted odds ratio (aOR) of 7.82 (95% CI 2.46–24.87; *p* < 0.001). Age showed no association with the outcome (aOR per year 0.99; 95% CI 0.95–1.04; *p* = 0.728). None of the baseline mobility categories reached statistical significance after adjustment, with aORs ranging from 0.37 for “walks with technical aid” (95% CI 0.12–1.15; *p* = 0.086) to 1.20 for “walks with major assistance” (95% CI 0.18–8.28; *p* = 0.851) versus independent ambulation as reference. For the ASA classification categories, standard errors were extremely large, yielding non-informative confidence intervals; this pattern is consistent with quasi-complete separation in the reference category (ASA I, n = 3), and therefore the corresponding effect estimates should not be interpreted as reliable adjusted associations. The Results are presented in Table 3.

Overall, after adjustment for age, ASA classification, and baseline mobility status, patients with an explicit medical order for mobilization had approximately 7.8-fold higher odds of being mobilized within the first 24 h after surgery than those without such an order. This organizational factor emerged as the strongest—and only statistically robust—independent determinant of compliance with the early-mobilization quality indicator in our cohort.

### 3.7. Significant Associations

In contrast, statistically significant associations were identified between mobilization within the first 24 h and the following variables: blood transfusion, performance of postoperative radiography within 24 h, and the existence of an explicit order to mobilize.

Patients who received blood transfusions had a lower probability of early mobilization compared with non-transfused patients (33.3%, 6/18 vs. 71.1%, 86/121), representing an absolute reduction of 37.7 percentage points (OR 0.203; 95% CI 0.07–0.58; *p* = 0.002).

Conversely, the performance of postoperative radiography within the first 24 h was associated with a higher frequency of early mobilization (69.8%, 88/126 vs. 30.8%, 4/13), with an OR of 5.29 (95% CI 1.53–18.3; *p* = 0.004).

The explicit order to mobilize showed the strongest association. Patients with this indication had an early mobilization rate of 72.5% (87/120), compared with 26.3% (5/19) among those without the order (OR 7.38; 95% CI 2.46–22.11; *p* < 0.001).

## 4. Discussion

This observational study shows that hip fractures occur more frequently in older patients and are more prevalent in women than in men.

In line with these findings, international data indicate that the incidence of this condition increases markedly after the age of 70, peaking in individuals over 80 years old, with between 70% and 75% of cases occurring in women [12,13]. These data reinforce that advanced age and female sex remain the main structural risk factors for hip fracture worldwide.

According to the Barthel Index results (51.8%), more than half of the participants maintained an adequate level of independence in basic activities of daily living prior to surgery. This trend is consistent with findings from recent epidemiological studies showing that a relevant proportion of adults undergoing hip fracture surgery preserve some degree of functional autonomy even at advanced ages [14,15].

Similarly, the comparable proportion of cognitive impairment observed among participants aligns with reports by Cieza-Macedo et al. and Coviello et al. [16,17].

Regarding mobility, the data show a less favorable situation: more than half of the participants presented some degree of limitation (55.4%), and one-third required assistive devices (33.1%). Recent literature highlights that mobility problems increase substantially after the age of 75, even in the absence of cognitive impairment [18,19]. Furthermore, approximately one-third of patients require assistive devices prior to hip fracture due to musculoskeletal frailty and progressive loss of strength, which is consistent with the findings of this study [20].

The distribution of patients according to the ASA classification revealed that most of the sample had a highly compromised clinical profile, characterized by a high burden of disease and considerable anesthetic risk. This pattern is consistent with what is commonly observed in medium- and high-complexity hospital settings, where patients frequently present multiple chronic conditions requiring individualized perioperative management [21].

However, our findings do not confirm the close relationship between preoperative physical status, as measured by ASA classification, and the likelihood of achieving early mobilization after hip fracture surgery, contrary to other studies in which ASA has been identified as a predictor of early mobilization probability [22].

Early mobilization in our study was influenced by several patient-related factors, including the need for blood transfusion, which was present in 12.9% of patients (n = 18). These rates are lower than those reported in other studies, where transfusion rates range between 30% and 40% [23,24].

However, when analyzing the relationship between transfusion and postoperative weight-bearing authorization, differences emerge. In the study by María Macho [24], statistical significance was not reached, with 74.2% of transfused patients authorized for postoperative weight-bearing and 25.8% maintained without load-bearing. In contrast, our findings showed that 66% of transfused patients were not authorized for weight-bearing compared with 33% who maintained load-bearing.

In recent years, the concept of Patient Blood Management (PBM) has emerged in the surgical patient setting and could be applicable to hip fracture management. The fundamental pillar of PBM consists of optimizing hemoglobin levels through hematinic precursors (iron, vitamin B12, and folic acid), together with erythropoietin administration, enabling patients to reach surgery with adequate hemoglobin levels and potentially avoid perioperative transfusion [25,26,27,28,29].

Hemodynamic instability [30], postoperative delirium—manifested as hyperactive, hypoactive, or mixed subtypes [31]—and resistance to mobilization also represent major challenges, often accompanied by patient reluctance to mobilize [32].

The presence of acute stress and post-traumatic stress disorder after surgery may also influence recovery. Orthopedic and trauma surgeons tend to focus primarily on the physical aspects of treatment, whereas rehabilitation adopts a more holistic perspective, considering the individual as a whole [33].

Therefore, treatment and rehabilitation should focus not only on restoring physical function but also on supporting psychological and social reintegration. Early mobilization should thus be accompanied by psychological support [34].

Another perioperative factor amenable to intervention is pain, which perpetuates the immobility cycle. Analgesia is often required to facilitate mobilization, highlighting the importance of integrating pre-planned pharmacological interventions to achieve optimal pain control, in coordination with nursing staff [22].

This aspect, together with the prescription of postoperative radiography within 24 h and the explicit order to mobilize, represents organizational factors that can be addressed through the implementation of an early mobilization protocol, formally initiated by medical prescription [33,35].

Such a protocol constitutes a promising, cost-effective, and patient-centered intervention to improve recovery outcomes.

These findings should be interpreted with caution, given the retrospective design of the study and the absence of fracture-specific and surgery-related variables, which are known to influence postoperative mobilization.

## 5. Strengths and Limitations

This study has several strengths. It provides real-world data from a clinical setting and focuses on a relevant and actionable quality indicator in hip fracture care.

However, several limitations should be acknowledged. First, the retrospective and single-center design may limit generalizability. Second, fracture-specific and surgery-related variables were not available, which may confound the interpretation of the results. Third, the final sample size was slightly lower than initially estimated. Fourth, blood transfusion was used as an indirect marker of clinical status. Finally, a substantial proportion of cases lacked documented reasons for non-mobilization, which may affect the robustness of the findings.

An additional methodological limitation of the multivariable model is the quasi-complete separation observed in the ASA classification variable, most likely related to the small number of patients in the reference category (ASA I, n = 3). This precluded reliable estimation of adjusted odds ratios for the ASA categories; nonetheless, bivariate analysis did not suggest a meaningful association between ASA class and early mobilization (*p* = 0.431), which supports the overall conclusion that ASA physical status was not a main determinant of compliance in our cohort.

## 6. Conclusions

This study highlights that early mobilization after hip fracture surgery is not solely determined by patient-related factors but is strongly influenced by modifiable clinical and organizational processes embedded within routine care. In particular, the presence of an explicit medical order to mobilize emerged as the most relevant independent factor, reinforcing the critical role of structured care pathways and interdisciplinary coordination.

These findings suggest that early mobilization should be conceptualized not only as a patient-dependent outcome but also as a system-level quality indicator, reflecting the efficiency, coordination, and responsiveness of postoperative care. From this perspective, variability in mobilization rates may be more closely related to differences in care processes than to intrinsic patient characteristics.

However, caution is warranted in interpreting these results. The absence of fracture-specific and surgery-related variables, including fracture subtype, fixation stability, surgical technique, and postoperative weight-bearing protocols, represents an important limitation and may partially explain the observed associations.

Despite these limitations, our results provide actionable insights for clinical practice. The implementation of standardized protocols, automatic mobilization orders integrated into electronic medical records, and streamlined postoperative workflows may represent low-cost, high-impact interventions to improve adherence to early mobilization targets.

Future research should focus on prospective, multicenter designs integrating surgical, physiological, and organizational variables to better define the relative contribution of each domain and to establish robust, evidence-based strategies for optimizing postoperative recovery in patients with hip fractures.

## 7. Future Directions

Future research should focus on the prospective evaluation of protocolized early mobilization pathways in patients undergoing hip fracture surgery, assessing their impact on functional recovery, postoperative complications, and length of hospital stay.

Multi-center studies would be valuable to validate the organizational and clinical determinants identified in this study and evaluate variability across different healthcare settings.

In addition, interventional designs assessing the implementation of automatic mobilization orders, standardized postoperative radiography circuits, and transfusion optimization strategies could provide higher-level evidence regarding their effectiveness in improving early mobilization compliance.

Further research should also explore the role of multidisciplinary educational interventions aimed at healthcare professionals, as well as patient-centered strategies to enhance participation in postoperative mobilization.

Finally, integrating digital alert systems and postoperative checklists within electronic medical records represents a promising area for quality improvement research, particularly in relation to adherence monitoring and real-time clinical decision support.

## Figures and Tables

**Table 1:** Functional and mobility levels.

Variables	n = 139	(%)
Pfeiffer Scale (Cognitive)		
0 (None)	72	51.8
1 (Mild)	29	20.9
2 (Moderate)	18	12.9
3 (Severe dependence)	20	14.4
Barthel Index (Dependence)		
0 (Independent)	72	51.8
1 (Moderate dependence)	41	29.5
2 (Severe dependence)	23	16.5
3 (Total dependence)	3	2.2
Physical Mobility Scale		
0 (Independent)	38	27.3
1 (Walks with difficulty)	31	22.3
2 (Technical aid required)	46	33.1
3 (Major assistance)	9	6.5
4 (Extreme difficulty)	11	7.9
5 (Bed–chair bound)	4	2.9

**Table 2:** Factors associated with early mobilization within the first 24 h.

Variable	Mobilized < 24 h (n = 92)	Not Mobilized < 24 h (n = 47)	*p*-Value
Blood transfusion	6 (6.5%)	12 (25.5%)	0.002
No transfusion	86 (93.5%)	35 (74.5%)	
Postoperative radiography < 24 h	88 (95.7%)	38 (80.9%)	0.004
No radiography < 24 h	4 (4.3%)	9 (19.1%)	
Mobilization order	87 (94.6%)	33 (70.2%)	<0.001
No mobilization order	5 (5.4%)	14 (29.8%)	

**Table 3:** Multivariable logistic regression analysis of factors associated with early mobilization within the first 24 h after hip fracture surgery.

Variable	β (SE)	aOR	95% CI	*p*-Value
Age (per year)	−0.008 (0.023)	0.99	0.95–1.04	0.728
Mobilization order (Yes vs. No)	2.057 (0.590)	7.82	2.46–24.87	<0.001
ASA classification (ref.: ASA I)				
ASA II	−14.52 (1382.9)	– ^*a*^	– ^*a*^	0.992
ASA III	−14.82 (1382.9)	– ^*a*^	– ^*a*^	0.991
ASA IV	−15.34 (1382.9)	– ^*a*^	– ^*a*^	0.991
ASA V	0.129 (2769.5)	– ^*a*^	– ^*a*^	1.000
Unknown	0.129 (2769.5)	– ^*a*^	– ^*a*^	1.000
Baseline mobility (ref.: independent)				
Walks with difficulty	−0.635 (0.621)	0.53	0.16–1.79	0.307
Walks with technical aid	−1.002 (0.583)	0.37	0.12–1.15	0.086
Walks with major assistance	0.185 (0.984)	1.20	0.18–8.28	0.851
Severe difficulty	−0.398 (0.873)	0.67	0.12–3.72	0.648
Bed–chair bound	−0.496 (1.263)	0.61	0.05–7.24	0.695

β, regression coefficient; SE, standard error; aOR, adjusted odds ratio; 95% CI, 95% confidence interval. ^*a*^ Adjusted odds ratios and 95% confidence intervals for ASA categories are not reported because the extremely large standard errors (≈1383) denote quasi-complete separation in the reference category (ASA I, n = 3), yielding non-informative point estimates and confidence intervals ranging from <$10^{-10}$ to >$10^{10}$.

## Data Availability

The data that support the findings of this study are available from the corresponding author upon reasonable request, subject to data protection regulations.

## Abbreviations

The following abbreviations are used in this manuscript:

FAC	Functional Ambulation Classification
ADL	activities of daily living
LOS	Length of stay
RNFC	Registro Nacional de Fracturas de Cadera
